# Terminal fucose mediates progression of human cholangiocarcinoma through EGF/EGFR activation and the Akt/Erk signaling pathway

**DOI:** 10.1038/s41598-019-53601-8

**Published:** 2019-11-21

**Authors:** Somsiri Indramanee, Kanlayanee Sawanyawisuth, Atit Silsirivanit, Paweena Dana, Chatchai Phoomak, Ryusho Kariya, Nathakan Klinhom-on, Supannika Sorin, Chaisiri Wongkham, Seiji Okada, Sopit Wongkham

**Affiliations:** 1 Department of Biochemistry, Faculty of Medicine, Khon Kaen University, Khon Kaen, 40002 Thailand; 2 Cholangiocarcinoma Research Institute, Khon Kaen University, Khon Kaen, 40002 Thailand; 3 Center for Translational Medicine, Khon Kaen University, Khon Kaen, 40002 Thailand; 4 Division of Hematopoiesis, Joint Research Center for Human Retrovirus Infection, Kumamoto University, Kumamoto, Japan

**Keywords:** Glycoconjugates, Bile duct cancer

## Abstract

Aberrant glycosylation is recognized as a cancer hallmark that is associated with cancer development and progression. In this study, the clinical relevance and significance of terminal fucose (TFG), by fucosyltransferase-1 (FUT1) in carcinogenesis and progression of cholangiocarcinoma (CCA) were demonstrated. TFG expression in human and hamster CCA tissues were determined using *Ulex europaeus* agglutinin-I (UEA-I) histochemistry. Normal bile ducts rarely expressed TFG while 47% of CCA human tissues had high TFG expression and was correlated with shorter survival of patients. In the CCA-hamster model, TFG was elevated in hyperproliferative bile ducts and gradually increased until CCA was developed. This evidence indicates the involvement of TFG in carcinogenesis and progression of CCA. The mechanistic insight was performed in 2 CCA cell lines. Suppression of TFG expression using siFUT1 or neutralizing the surface TFG with UEA-I significantly reduced migration, invasion and adhesion of CCA cells in correlation with the reduction of Akt/Erk signaling and epithelial-mesenchymal transition. A short pulse of EGF could stimulate Akt/Erk signaling via activation of EGF-EGFR cascade, however, decreasing TFG using siFUT1 or UEA-I treatment reduced the EGF-EGFR activation and Akt/Erk signaling. This evidence provides important insight into the relevant role and molecular mechanism of TFG in progression of CCA.

## Introduction

Glycosylation is a posttranslational modification of proteins that play pivotal roles in many physiological functions such as cell signaling, cell recognition and cell-cell interactions. The sugar moieties of the membranous glycoproteins confer structural variability and binding-specificity to their binding partners. The biosynthesis of glycan branches is not a template-based process as are DNA, RNA or proteins. It is, however, driven by a set of glycosylation machinery, e.g., glycosyltransferases and glycosidases, sugar transporters and activated sugar donors. According to this molecular basis, biosynthesis of glycans generates the molecular microheterogeneity and complexity of glycan which is associated with many physiological and pathological events. Aberrant glycosylation is often shown to be associated with the acquisition of all cancer hallmarks, i.e., cell growth, migration, invasion, cell-cell and cell-matrix interactions, immune modulation, angiogenesis and metastasis^1^.

Incomplete synthesis and neo-synthesis are the two postulated processes resulting in the alterations of tumor-associated membranous glycan structures^2^. The incomplete synthesis of glycan structures such as sialyl Tn (STn) is frequently reported in early stages and associated with cancer cell invasion^3,4^. On the other hand, neo-synthesis of glycans, e.g., Sialyl- Lewis (SLe^a^) and SLe^x^ are commonly observed in advanced stages and associated with cancer metastasis^5^. Modification of adhesion molecules such as E-cadherin and integrin has also been found to be associated with cancer progression. E-cadherin with β1, 6-N-acetylglucosamine-branched N-glycan structures decreased cell-cell adhesion and promoted cell dissociation and invasion of gastric cancer cells^6^.

Fucosylation is a process of adding a single fucose to the reducing end of a glycan structure. It mediates several specific biological activities and contributes to several abnormal characteristics of cancer cells. Fucosylation is catalyzed by fucosyltransferases (FUTs). At present, 13 FUTs catalyze the reaction with different linkages to oligosaccharides, yielding different glycan structures on glycoconjugates. FUT1 catalyzes the transfer of a fucose residue to the terminal galactose of lactosamine precursor type II whereas FUT8 transfers a fucose to the innermost N-acetylgalactosamine. Aberrant expressions of FUTs and fucosylated glycans occur in association with advanced tumor stages and poor prognosis has been reported in various cancers^7^. Understanding of fucosylation in a particular cancer may provide a clue to discover an early diagnosis and possibly a novel treatment.

Cholangiocarcinoma (CCA) is a malignancy of bile duct epithelia along with any part of the biliary tree. Although CCA is rare worldwide, the global incidence is now increasing^8^. The highest incidence of CCA had been reported in the Northeast of Thailand where liver fluke (*Opisthorchis viverrini*, Ov) infection is known to be a major risk factor^8,9^. As CCA has a silent clinical development and currently there is no early tumor marker for diagnosis, most CCA patients are identified at an advance stage and die of metastasis^10^. A better understanding of the mechanism underlying metastasis may lead to a novel target therapy for the metastasized CCA.

The information of FUTs and fucosylated glycans in CCA are limited. The aberrant fucosylation in human CCA tissues was reported previously^11^. Using *Ulex europaeus* agglutinin-I (UEA-I), a lectin that recognizes the terminal α1, 2- fucose containing glycan (TFG)^12^, showed that TFG was not detected in normal bile duct epithelia but was highly expressed in hyperplastic/dysplastic bile ducts and CCA tissues^11^. In the present study, the clinical significance of the TFG in CCA was further demonstrated. The association and the molecular mechanism of TFG in carcinogenesis and progression of CCA were also shown. Moreover, the role of TFG on EGF-EGFR activation was underscored.

## Results

### TFG was over-expressed in hyperplastic bile duct epithelia and CCA tissues

**Figure 1:**
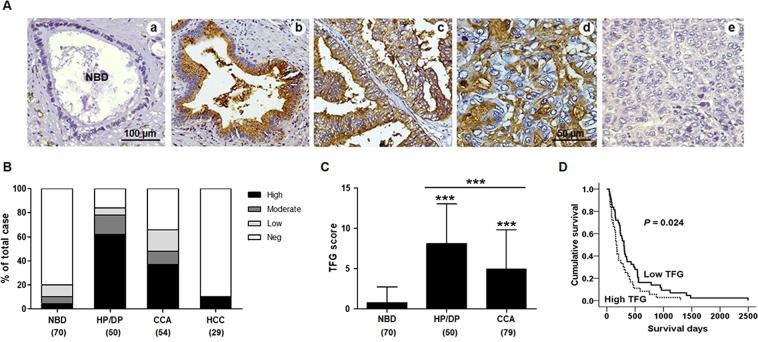
TFG expression in tumor tissues of CCA patients. (**A**) The reactivity of TFG in tumor tissues of CCA and hepatoma was determined using UEA-I histochemistry. (a) Normal bile ducts showed negative TFG signals, whereas (b) hyperplastic/dysplastic bile ducts, (c) papillary type- and (d) non-papillary type CCA showed positive TFG expressions. (e) Hepatoma did not express TFG. Original magnification; a–c x200 and d,e x400. Scale bar = 100 μm for (a–c), and = 50 μm for (d,e). (**B**) Differential expressions of TFG found in normal bile duct (NBD), hyperplastic/dysplastic bile ducts (HP/DP), CCA and hepatoma (HCC). Data are presented as a percentage of total cases. (**C**) The expression levels of TFG (mean ± S.D.) in HP/DP bile ducts and CCA tissues were significantly elevated compared with those of normal bile ducts. ***P < 0.001; Student’s *t*-test. (**D**) Kaplan-Meier analysis and Log-rank tests showing a significant correlation of CCA patients with high TFG and short survival (*P* = 0.024).

TFG in 79 paraffin-embedded human CCA tissues was determined using UEA-I histochemistry. As shown in Fig. 1Aa, normal bile duct epithelia and hepatocytes were mostly negative for TFG. In contrast, the positive TFG signal with suprabasal staining in cytoplasm and stratification of nuclei were observed in the hyperplastic/dysplastic bile ducts (Fig. 1Ab). For cancerous tissues, CCA of the papillary type displayed cytoplasmic focal apical staining of TFG and a variation of nuclear feathers (Fig. 1Ac), whereas CCA of the non-papillary type mostly showed cytoplasmic staining of TFG with irregular nuclei (Fig. 1Ad). In addition, Kuffer’s cells, red blood cells and endothelial cells were also positive for TFG.

CCA patients could be divided according to the TFG scores in tumor tissues into 4 groups; negative = 0, low = 1–3, moderate = 4–6 and high = 8–12. Most normal bile duct epithelia (80%) at the adjacent tissues to the tumor had negative signals for TFG, whereas hyperplastic/dysplastic bile ducts (84%) and CCA tissues (66%) had positive TFG signals (Fig. 1A,B). The TFG scores were significantly elevated in hyperplastic/dysplastic bile ducts and CCA compared with those of normal bile ducts (*P* < 0.001; Fig. 1C). In contrast to CCA, most of hepatocellular carcinoma tissues (26/29, 89.6%) were negative for TFG staining (Fig. 1Ae,B). The association of numbers of infiltrating cells and TFG scores in CCA tissues was also determined. A majority of CCA patients (70%) had a low number of infiltrating cells and a minority (30%) had medium to high infiltrating cells numbers. The association between infiltrating cells and TFG scores, however, was not observed.

### A high TFG score was an independent prognostic factor for poor survival of CCA patients

To signify the association between TFG expression and clinical features of CCA patients, a univariate analysis was applied. CCA patients were divided according to the median of TFG scores into low (TFG = 0–4, n = 43) and high (TFG = 6–12, n = 37). The stratification did not lead to any significant correlation of TFG expression with clinical features of age, gender, histotype, tumor size, regional lymph node metastasis and staging of the patients (Table S1). To further explore the clinical relevance of TFG on survival of CCA patients, a Kaplan-Meier plot and Log rank test were performed. The median survival time of CCA patients with a high TFG score was 167 days (95% confidence interval, CI; 132–202 days) and those with a low TFG was 302 days (95% CI; 247–357 days). As shown in Fig. 1D, CCA patients with high a TFG had a significantly shorter survival than those with a low TFG (*P* = 0.024). In addition, multivariate analysis using Cox-proportional hazard regression suggested the significance of TFG (*P* = 0.044) and histological types (*P* = 0.008) as independent prognostic factors for poor survival time of CCA patients (Table 1).

**Table 1:** Multivariate analysis of TFG expression in patients with cholangiocarcinoma.

Variables	n	Adjusted HR	95% CI	*P*-value
TFG score				
<6	43	1	1.012–2.592	0.044
≥6	36	1.620		
Age				
≤56 years	41	1	0.619–1.586	0.969
>56 years	38	0.991		
Sex				
Male	54	1	0.675–1.816	0.688
Female	25	1.107		
Histological type				
Papillary type	25	1	1.210–3.621	0.008
Non-Papillary type	54	2.093		
Tumor stage				
I–III	30	1	0.717–1.861	0.554
IVA and IVB	49	1.155		

### High expression of TFG related to carcinogenesis of CCA in hamster model

**Figure 2:**
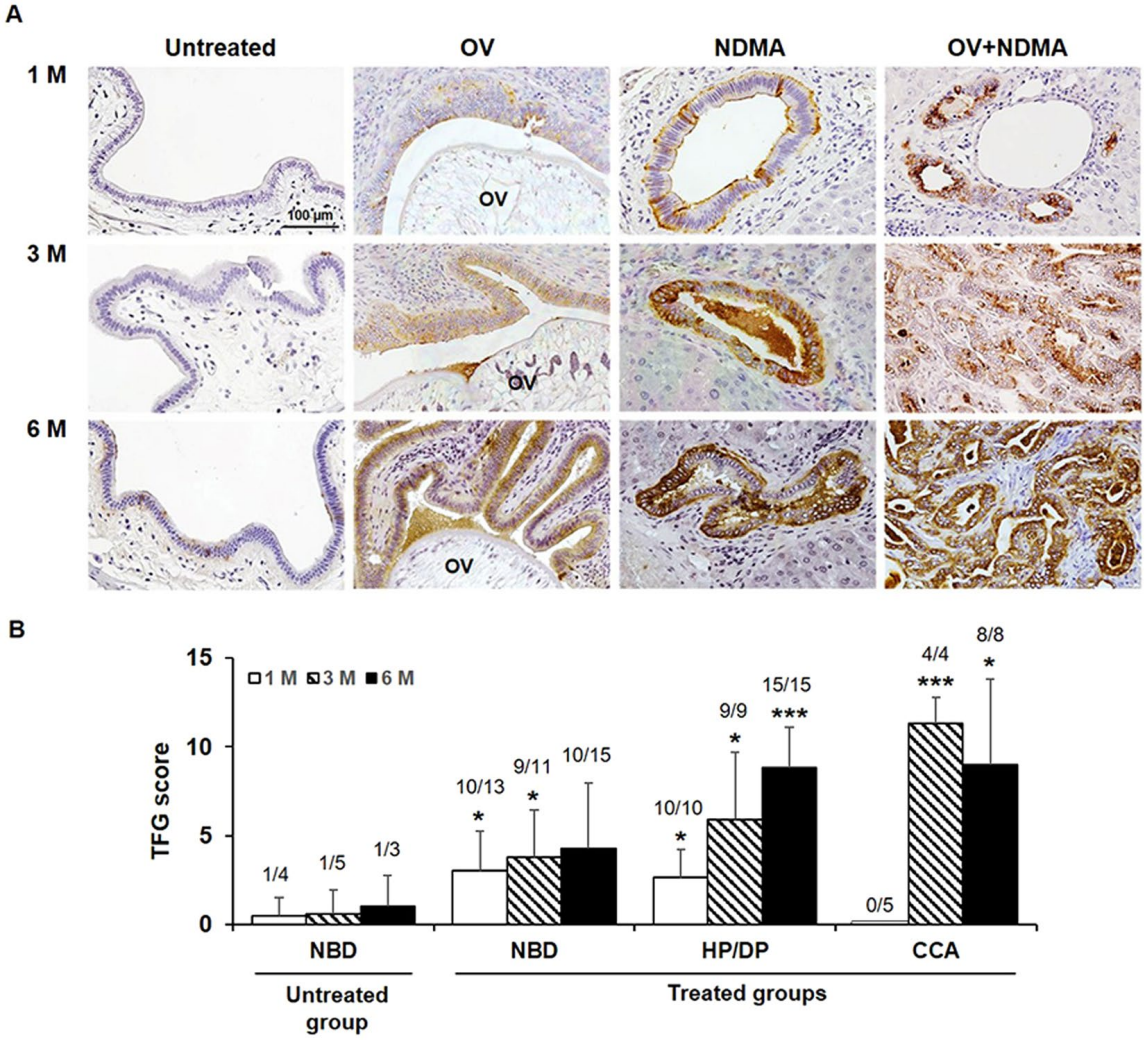
The expression profile of TFG in liver sections of hamsters. (**A**) Hamsters were divided into 4 groups of the Ov-associated CCA hamster model: untreated control, Ov infected, NDMA treated and Ov + NDMA (CCA). Liver sections were obtained from each group at 1, 3 and 6-month post-treatment. The reactivity of TFG in tissues was determined using UEA-I histochemistry. TFG signals were undetectable in normal bile ducts but were observed in every treatment group and as early as 1-month post-treatment. Original magnification; x 200. Scale bar = 100 μm. (**B**) The TFG signal was quantitated according to the Allred scoring system. TFG scores were analyzed according to the pathology of the bile duct epithelia as normal (NBD), hyperplastic/dysplastic bile ducts (HP/DP), and CCA observed at each time point. Data are mean ± S.D. Numbers indicate the number of animals that had positive TFG/total numbers. *P < 0.05 and ***P < 0.001; Student’s *t*-test.

The Ov + NDMA-induced CCA hamster model closely mimics the pathogenesis of human CCA^13^ and hence was used to study the association of TFG with carcinogenesis of CCA in this study. NDMA^14^ is demethylated by hepatic microsomes to yield genotoxic reactive intermediates. Alkylation of DNA by these reactive metabolites results in a change in base sequence and deletion of one or more base pairs leading to the NDMA-induced carcinogenesis^14^. The UEA-I histochemistry of liver tissues from 4 groups of hamsters including untreated, Ov infected, NMDA treated and Ov + NDMA treated (CCA) groups at 1, 3 and 6 months post-treatment was performed. All treated groups exhibited bile duct epithelia with hyperplasia/dysplasia after treatment. In the NDMA treated group, CCA was found in 3/5 of the hamsters at 6-months post-treatment. In the Ov + NDMA treated group there was no CCA developed at 1 month, however, 4/5 hamsters in 3-months and 5/5 in 6-months post-treatment group developed CCA. No inflammation was found in the untreated-control group; however, the infiltrating cells were found in all treated groups but had no association with TFG scores. Normal bile ducts of the non-treated group were rarely positive for TFG whereas all hyperplastic/dysplastic bile ducts and the CCA ducts from the treated group were positive for TFG with differential signals (Fig. 2A). TFG reactivity was detected in the bile duct epithelia from all treated groups as early as 1-month post-treatment and the signal increased progressively with time of treatment. The TFG signals of each histological subtype of bile duct epithelia from all treated groups were quantitatively analyzed as shown in Fig. 2B.

### Suppression of TFG expression significantly decreased progression of CCA cells

**Figure 3:**
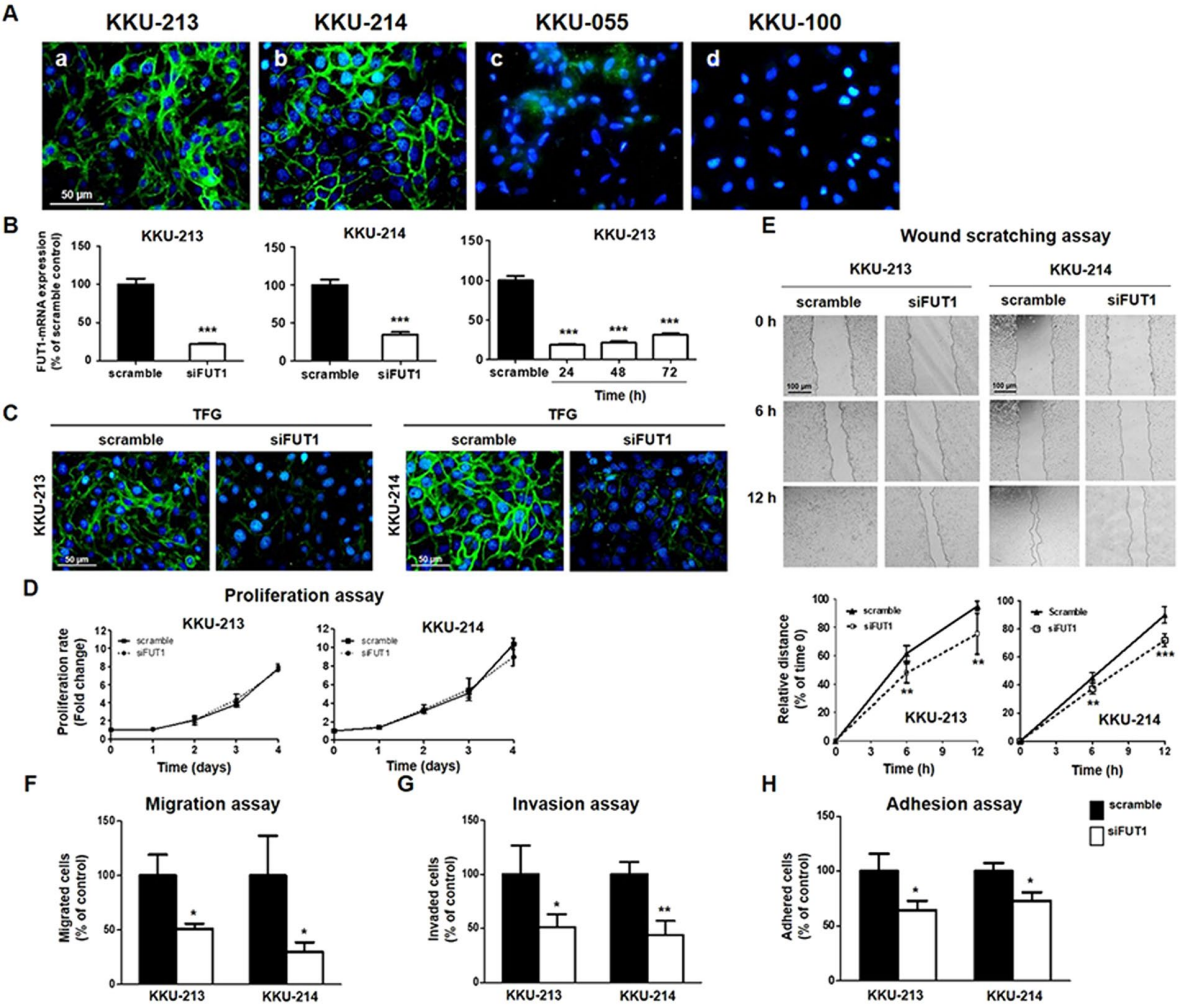
Suppression of TFG signals using siFUT1 significantly decreased metastatic ability of CCA cells. (**A**) TFG differentially expressed in 4 CCA cell lines as determined by UEA-I cytofluorescent staining. Scale bar = 50 μm (**B**) siFUT1 significantly decreased the expression level of FUT1 mRNA in KKU-213 and KKU-214 cells and the effect was apparent even at 72 h. (**C**) UEA-I cytofluorescent staining indicates suppression of TFG expression in siFUT1 treated cells compared to the scramble control cells. Scale bar = 50 μm. (D) Suppression of FUT1 had no effect on cell proliferation of CCA cells. (**E**) wound scratching, (**F**) migration, (**G**) invasion and (**H**) adhesion of siFUT1 treated cells were reduced as compared to those of the scramble controls. Scale bar = 100 μm. All assays were performed in triplicate and the data represented are mean ± SD from one of two independent experiments. *P < 0.05, **P < 0.01, ***P < 0.001, Student’s *t*-test.

The endogenous expression levels of TFG in 4 CCA cell lines were first determined by UEA-I cytofluorescent staining. CCA cell lines differentially expressed TFG (Fig. 3A); KKU-213 and KKU-214 had strong UEA-I signals whereas KKU-055 and KKU-100 showed weak signals. CCA cells with high TFG expression were selected for further elucidating the functional roles of TFG in CCA cells.

The specific siRNA to FUT1, the enzyme responsible for TFG biosynthesis, was used to suppress TFG expression. siFUT1 effectively suppressed FUT1 mRNA expression of KKU-213 and KKU-214 cells to 30–40% of the scramble controls and the effect lasted until 72 h (Fig. 3B, *P* < 0.001). Suppression of FUT1 by siRNA significantly reduced TFG expression as shown by the reduction of TFG signals in UEA-I cytofluorescent stained cells (Fig. 3C). The effect of TFG on cell proliferation was first examined. As shown in Fig. 3D, treatment of siFUT1 had no effect on cell proliferation of either KKU-213 or KKU-214 cells as observed for 96 h. Suppression of TFG expression using siFUT1, however, significantly reduced cell migration as shown in the wound scratching assay (*P* < 0.01) and migration assay (*P* < 0.05) (Fig. 3E,F). siFUT1 treatment also significantly decreased numbers of invaded and adherent cells to approximately 30–50% of the scramble control cells (Fig. 3G,H). These results suggested the contribution of TFG in progressive phenotypes of CCA cells.

### Cell surface TFG implicated the migration and invasion ability of CCA cells

**Figure 4:**
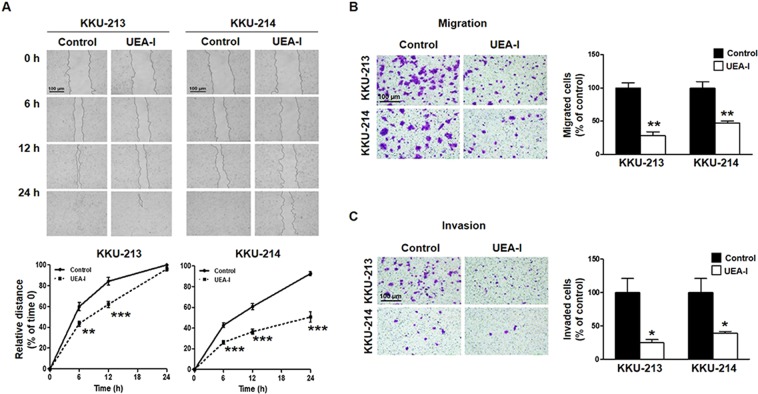
Masking cell surface TFG with UEA-I potently inhibited CCA cell migration and invasion. (**A**) Wound scratching assays of KKU-213 and KKU-214 were performed in the presence (UEA-I) or absence (control) of 5 µg/ml UEA-I and observed for 24 h. (**B**) migration and (**C**) invasion assays were performed in the presence of 5 µg/ml UEA-I, 9 h for KKU-213 and 24 h for KKU-214. Scale bar = 100 μm. All quantitative data are mean ± SD of triplicate assays from one of the two independent experiments. *P < 0.05, **P < 0.01, ***P < 0.001, Student’s *t*-test.

TFG was shown to locate at the cell membrane as illustrated in UEA-I histochemistry of CCA tissues and UEA-I cytofluorescent stained CCA cells. Inhibition of TFG function by masking cell surface TFG using UEA-I was designed. As lectin can cause cell aggregation, the concentration of UEA-I that did not generate cell aggregation was first optimized. KKU-213 was treated with various concentrations (0.78 µg/ml –100 µg/ml) of UEA-I for 1 h and the formation of aggregated cells was observed under a microscope. As shown in Fig. S1A, aggregated cells were first observed at 12.5 µg/ml UEA-I and no cell aggregations were detected at 6.25 µg/ml UEA-I, therefore, 5 µg/ml UEA-I was used for cell treatment. No effect of cell aggregation was found under this treatment for 24 h (Fig. S1B). Wound scratching assays were performed in the presence or absence of 5 µg/ml UEA-I. As compared to the controls, UEA-I treatment significantly reduced migration ability of both KKU-213 and KKU-214 cells (Fig. 4A, *P* < 0.001). CCA cells treated with 5 µg/ml UEA-I for 24 h were subjected to Boyden chamber migration and invasion assays. UEA-I treatment significantly decreased the migration ability of KKU-213 and KKU-214 to approximately 30–40% of the controls (Fig. 4B, *P* < 0.01). Similar effects were observed for invasion assays (Fig. 4C, *P* < 0.05). These results indicated the significance of surface TFG on migration and invasion of CCA cells.

### TFG mediated progression of CCA cells via activation of Akt and Erk

**Figure 5:**
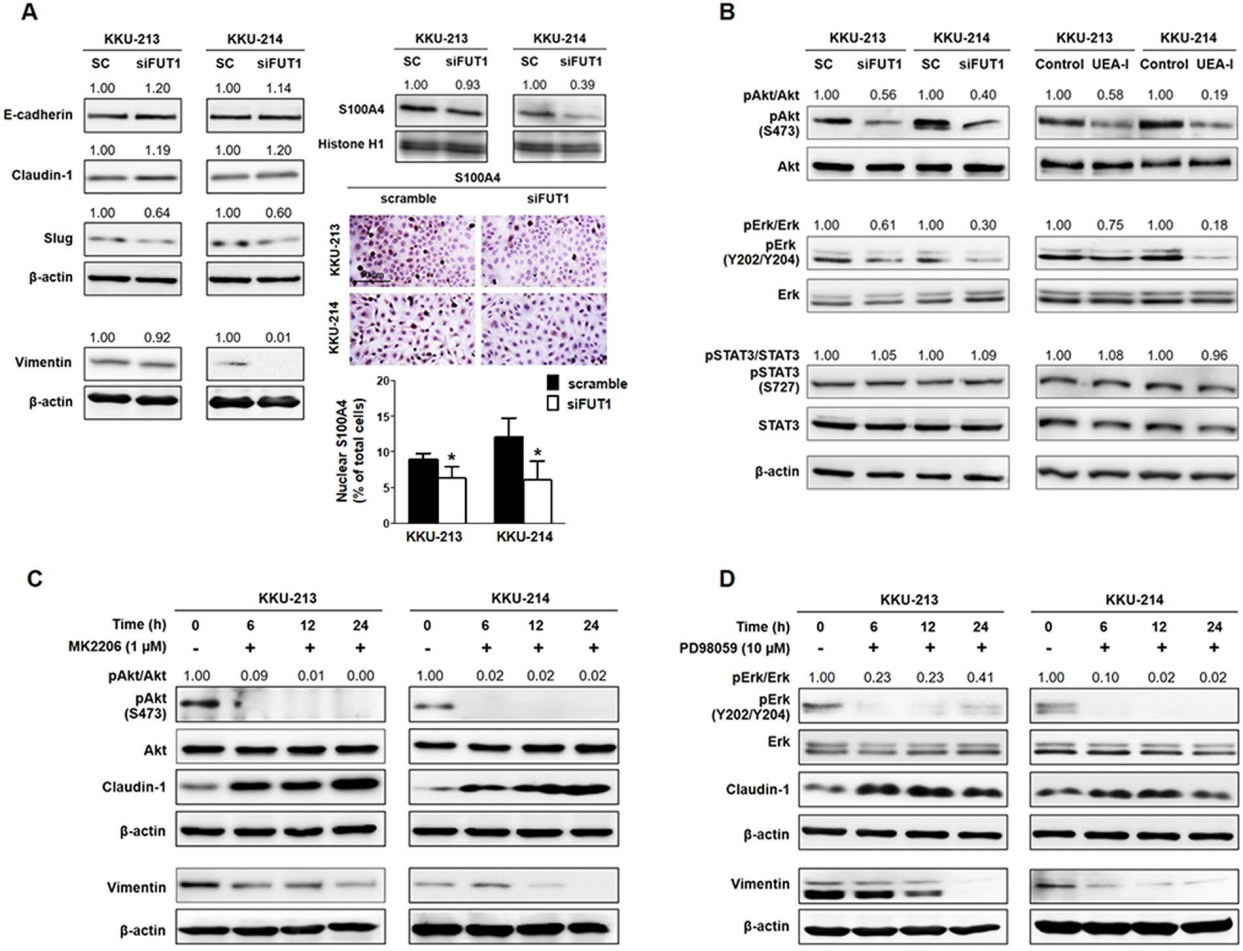
TFG modulated metastatic abilities of CCA cells via stimulation of Akt/Erk and EMT. (**A**) Epithelial-mesenchymal transition (EMT) markers: E-cadherin, claudin-1, slug, vimentin and nuclear S100A4 were determined using Western blotting in siFUT1 treated cells in comparison with scramble control (sc) cells. Nuclear S100A4 of sc and siFUT1 treated cells were also compared using S100A4-cytochemistry stain. Scale bar = 100 μm. (**B**) The phosphorylation of Akt, Erk and STAT3 were demonstrated by Western blotting in siFUT1 and UEA-I treated cells. Activation of Akt, Erk and STAT3 of the treated cells were compared with those of the control cells by assigning control cells = 1. CCA cells were treated with (**C**) Akt inhibitor, MK2206 and (**D**) Erk inhibitor, PD98059 for 6, 12 and 24 h. Phosphorylated Akt and Erk together with claudin-1 and vimentin were determined using Western blotting. Quantification of each protein was analyzed using β-actin as an internal control and assigning control cells = 1. In general protein lysate of 20 µg/well was loaded, except protein lysate of 30 µg for KKU-213 and 50 µg for KKU-214 were loaded for determination of vimentin. The data shown are representatives of 3 independent experiments. The full-length blots are presented in Supplementary Fig. 2A–D.

Epithelial mesenchymal transition (EMT) is a process involved in driving metastatic phenotypes of cancer cells, e.g., by cell migration, and invasion. Whether TFG mediated progression of CCA cells involved EMT was next examined. KKU-213 and KKU-214 cells were treated with siFUT1 for 24 h and the expression of epithelial markers, E-cadherin and claudin-1, and mesenchymal markers, slug and vimentin, were determined using Western blots. As shown in Fig. 5A, E-cadherin and claudin-1 were increased while slug and vimentin were conversely decreased upon siFUT1 treatment. These results indicated TFG mediated migration and invasion of CCA cells via activation of EMT. In addition, expression of nuclear S100A4, a member of small Ca2+-binding proteins that is involved in invasiveness and metastasization of CCA cells^15,16^ was also reduced in siFUT1 treated cells.

As E-cadherin, claudin-1, slug and vimentin are effector molecules modulated via activation of Akt, Erk and STAT3 which have been shown to be associated with progressive phenotypes of CCA^17–21^, whether these signal transduction pathways connected TFG with progression of CCA cells was further explored. KKU-213 and KKU-214 cells were treated with siFUT1 or UEA-I for 24 h and phosphorylation of Akt, Erk and STAT3 were determined using Western blots. As shown in Fig. 5B, compared with the control cells, phosphorylation of Akt and Erk but not STAT3 were markedly reduced under UEA-I and siFUT1 treatments. Whether activations of Akt and Erk mediated EMT in KKU-213 and KKU-214 cells were further investigated. CCA cells were treated with 1 µM of MK2006, a specific inhibitor of Akt or 10 µM of PD98059, an Erk inhibitor for 6, 12 and 24 h. The treatment effectively inhibited phosphorylation of Akt and Erk through 24 h (Fig. 5C,D). Inhibition of Akt and Erk phosphorylation effectively increased the expression of claudin-1 and suppressed the expression of vimentin in a time-dependent manner. Effects of Akt and Erk inhibitors on the expression of E-cadherin and slug, however, were not consistently observed under this treatment (data not shown). These results implied that TFG promoted migration and invasion of CCA cells via transduction of Akt and Erk, and activation of EMT.

### TFG favored EGF-EGFR activation

**Figure 6:**
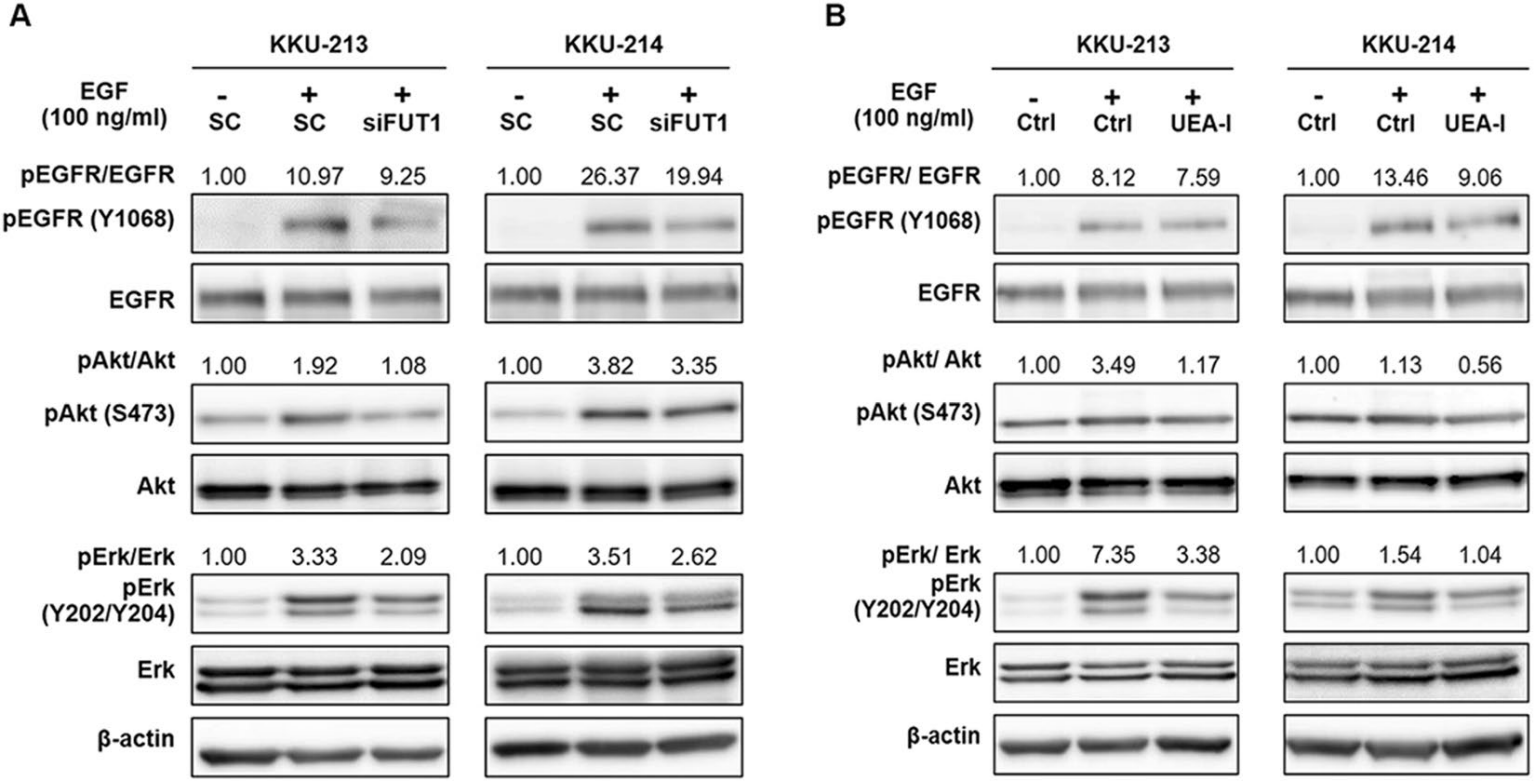
TFG favored EGF-EGRF activation. CCA cell lines were treated with (**A**) siFUT1 or scramble (sc) and (**B**) with UEA-I or without UEA-I (ctrl) for 24 h prior to the stimulation of EGF (100 ng/ml) in the serum free medium for 10 min. The levels of EGFR, phosphorylated EGFR and its downstream signaling Akt and Erk were determined using Western blotting. The activation of EGFR, Akt and Erk of the EGF treated cells were compared with those of the untreated cells by assigning the untreated cells = 1. The full-length blots are presented in Supplementary Fig. 3A,B.

EGF-EGFR has been documented to be an active signaling pathway in CCA^22–25^. As Akt and Erk are downstream effectors of EGFR activation^26,27^ and contribution of terminal fucose in EGF-EGFR interactions has been demonstrated^28–31^, therefore, TFG may favor EGF-EGFR activation and motivate Akt and Erk action. This postulation was next elucidated. Phosphorylation levels of EGFR at Y1068 as well as its downstream signaling, Akt and Erk, were used as the indicators of EGFR activation. KKU-213 and KKU-214 were treated with siFUT1 or 5 µg/ml UEA-I for 24 h prior to the stimulation of 100 ng/ml EGF in serum free medium for 10 min. The levels of phosphorylated EGFR, Akt and Erk, were determined using Western blotting. EGF treatment effectively activated EGFR action as the level of phosphorylated EGFR in EGF treated cells was higher than those of the untreated-control cells in both KKU-213 and KKU-214 (Fig. 6A). The phosphorylation level of EGFR, however, was reduced in siFUT1 treated cells. These results emphasized the positive involvement of TFG on EGF-EGFR activation. EGF treatment also activated Akt and Erk signaling, as phosphorylation levels of Akt and Erk were also increased in EGF treated cells and were decreased in siFUT1 treated cells. Similar results were observed in UEA-I treated cells (Fig. 6B). These results underscored the function of TFG on EGF-EGFR activation.

## Discussion

A body of evidence indicates specific changes of glycan biosynthesis pathways in cancer that may interfere with regulation of cell adhesion, migration and proliferation. Fucosylation and sialylation are the major glycosylation changes of typical terminal sugars that modify proteins and mediate vital biological functions in cancer^32–34^. This study reports for the first time, the significance of terminal fucose glycan, TFG, in tumor development and progression of CCA. Patients with high TFG expression in tumor tissues exhibited shorter survival than those with low TFG expression. The involvement of TFG in carcinogenesis was demonstrated in the liver fluke-induced CCA hamster model. The significance of TFG in promoting CCA progression was emphasized in this study. Suppression of TFG expression by siFUT1 or diminishing of TFG action using UEA-I significantly reduced CCA cell migration, invasion and adhesion. The mechanism underlying these observations was shown to be in part via EGFR, activation of Akt and Erk that consequently stimulated the EMT process. The results underscore the roles of TFG in progression of CCA.

Lectin-based histochemistry has been used to identify aberrant glycosylation in several tumor tissues. In the present study, we demonstrated the aberrant expression of TFG in hyperplasia/dysplasia of bile duct epithelia and tumor tissues of CCA patients and Ov-induced CCA hamsters using UEA-I histochemistry. The aberrant expression of TFG during tumor development was shown in Ov-induced CCA hamster models and high expression of TFG in bile duct epithelia with hyperplasia/dysplasia and CCA indicate the significance of TFG in CCA development and progression. High expression level of TFG in tumor seems to be a common feature of cancer as the similar observation was also reported for parotid gland mucoepidermoid carcinoma^35^, colon cancer^36^ and metastatic adenocarcinomas of lung^37^. Expression level of TFG did not relate with clinical features but had influence on survival of CCA patients. High expression of TFG in CCA tissues was an independent prognostic factor for short survival of patients. This observation is in agreement with the report in ovarian cancer^38^. Moreover, hepatocytes and HCC tissues rarely expressed TFG, thus TFG is possibly a marker for differential diagnosis between CCA and HCC.

The UEA-I histochemistry of hamster tissues from the Ov-induced CCA group (Ov + NDMA) revealed similar results as observed in CCA tissues of patients. The involvement of TFG in carcinogenesis of CCA was supported by the fact that TFG was rarely expressed in normal bile ducts but strongly expressed in hyperproliferative bile ducts and CCA at all stages of carcinogenesis. TFG may therefore be associated with pathogenesis and severity of pathogenesis of bile duct epithelia regardless of the etiology as aberrant expression of TFG was observed in hyperplastic/dysplastic bile duct epithelia found in all treated groups. Moreover, the expression level of TFG was gradually increased with time of treatment. Notably, TFG was observed as early as 1-month post-treatment before CCA development suggesting the significant role of TFG in early carcinogenesis and possibly as an early marker of CCA. These findings together support that TFG may play a significant role in pathogenesis of bile duct epithelia and carcinogenesis of CCA. These finding also raise the possibility of using TFG as a potential biomarker for the early detection of CCA. Additionally, aberrant expression of TFG may be a unique feature of CCA as TFG was detected in 84% of hyperplastic/dysplastic bile ducts and 66% in CCA but only 10% in HCC. This discrepancy suggests the possibility of using TFG as a differential marker between CCA and HCC liver cancers.

Fucosylation is an important feature that contributes to features of progressive phenotypes of tumor cells^7^ such as increased migration/invasion^39^, decreased adhesion^40^ and uncontrolled tumor growth^41^. siFUT1, the enzyme responsible for generating TFG, effectively decreased TFG expression in correspondence with the decrease of cell migration, invasion and adhesion, but not cell proliferation of CCA cells. Suppression of TFG expression by siFUT1 or TFG action by UEA-I treatment suppressed progressive phenotypes of CCA cells in a similar manner. These data provide important insights into the relevant role of TFG in progression of CCA and may explain the poor prognosis of CCA patients with high TFG levels reported in the current study. As the majority of CCA cases in this study were associated with Ov infection, the findings may not fully reflect those associated with other risk factors. The involvement of TFG and tumor metastasis, however, may depend on tumor origins. A positive correlation between TFG expression and lymph node metastases was demonstrated in lung metastatic adenocarcinomas^37^. In contrast, a high expression of TFG was correlated with decreased metastasis in mouth mucosal carcinomas^42^. These observations, however, connect the cell surface TFG and metastatic ability of CCA cells.

Current searches do not reveal reports on the mechanism underlying TFG promoted metastasis. A body of evidence, however, indicated the significance of Akt, Erk and STAT3 in regulation of cell migration and invasion in CCA cells^17–21^. Western blot analysis in the present study indicated that Akt and Erk but not STAT3 was inactivated in siFUT1 treated cells. This finding was confirmed by a UEA-I masking experiment. Epithelial-mesenchymal transition (EMT) is a well-known process related with an increase of cell motility and can be mediated via Akt and Erk activation^43–45^. Western blot analysis indicated that suppression of TFG expression reversed cells from mesenchymal to epithelial as the mesenchymal markers slug vimentin and nuclear S100A4 were decreased and the epithelial marker claudin-1 was increased in siFUT1 treated cells. Similar results were obtained in CCA cells treated with the specific inhibitor of Akt and Erk. Decreased phosphorylation of Akt and Erk was corroborated with Western blot analysis of increased claudin-1 and decreased vimentin. These findings together connect TFG to the activation of Akt and Erk and their downstream signal, EMT.

**Figure 7:**
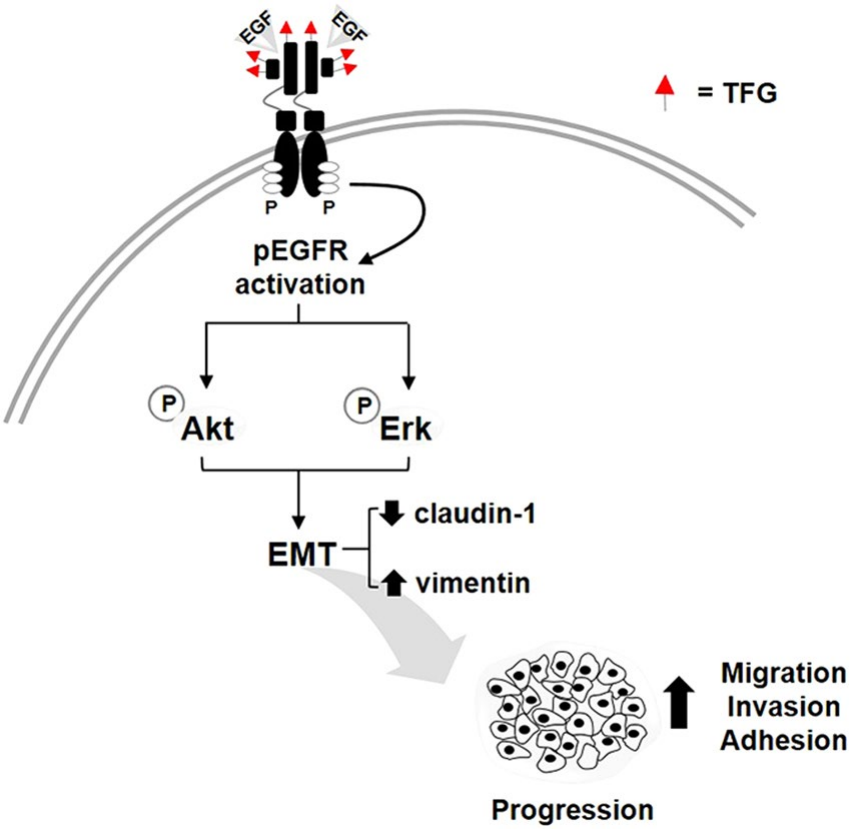
A model depicting the role of TFG mediates progression of CCA through the EGF/EGFR axis. TFG favors EGF-EGFR activation which stimulates its downstream signals by increasing the phosphorylation of Akt and Erk, and consequently enhances epithelial-mesenchymal transition by decreasing the epithelial marker (claudin-1) and increasing the mesenchymal marker (vimentin). The signal cascade promotes migration and invasion abilities of CCA cells.

Fucosylation affects the activity of tyrosine kinase receptors, especially EGFR. Activation of EGFR via ligand-induced dimerization leads to signaling cascade to activate Akt/Erk pathway^26,27^. Modification of EGFR by terminal fucose is necessary for ligand binding and intracellular signal transduction^28–31^. Overexpression of EGFR and its association to tumorigenesis and progression of CCA have been reported^22–25^. The present study illustrated the implication of TFG in EGFR activation and CCA progression through the stimulation of Akt/Erk and EMT. A short pulse of EGF markedly activated phosphorylation of EGFR and consequently stimulated Akt and Erk signaling. The activation of EGF, however was reduced when TFG expression was suppressed as shown in siFUT1 and UEA-I treated cells, resulting in the reduction of phosphorylated Akt and Erk. It has been suggested that TFG may facilitate EGF-EGFR binding by stabilizing electrostatic interactions^46^ leading to conformational changes of EGFR and EGFR autophosphorylation^47^. The overall mechanism of TFG in promoting progression of CCA is proposed as illustrated in Fig. 7.

## Conclusions

The overall results depicted the clinical significance of TFG in CCA development and progression. TFG may be an early marker for pathogenesis of the bile duct and a targeted molecule to improve CCA therapy. The findings in this study together signified TFG as a progression promoting factor in CCA. Activations of EGFR/Akt/Erk and induction of EMT were demonstrated to be an underlying mechanism.

## Materials and Methods

### Patient and hamster tissues

The paraffin-embedded liver tissues from 79 histologically proven CCA patients were obtained from the specimen bank of the Cholangiocarcinoma Research Institute, Faculty of Medicine, Khon Kaen University, Thailand. All patients in this study were classified as mass-forming with bile duct type (mucinous) intrahepatic CCA according to Banales *et al*.^9^. A majority of the cases (52 of 79, 66%) were non-papillary type CCA. All patients underwent curative intent surgery of CCA at Srinagarind hospital, Faculty of Medicine, Khon Kaen University, Thailand. Informed consent was obtained from each subject. The study design was approved by the Human Research Ethics Committee, Khon Kaen University (HE591063) and all research was performed in accordance with relevant guidelines and the Declaration of Helsinki.

The liver tissues from Ov-associated CCA hamsters were obtained as described previously^48^. The experimental hamsters were divided into 4 groups of 5 hamsters per group; the non-treated control, Ov infected, NDMA treated, and Ov + NDMA treated groups. Animals were euthanized at 1, 3, and 6 months post-treatment. UEA-I histochemistry was performed and compared between groups. The Animal Ethics Committee of Khon Kaen University approved this study (AEMDKKU1/2558) and all experiments were performed in accordance with relevant guidelines and regulations.

### Cell culture and treatments

All human CCA cell lines were established from primary tumors of Thai CCA patients with intrahepatic mass forming CCA^49^. KKU-055 was from a mixed histological type CCA; both well and poorly differentiated tubular adenocarcinoma, KKU-100 was from a poorly differentiated tubular adenocarcinoma, whereas KKU-213 and 214 were originated from adenosquamous carcinoma CCA. All cell lines were obtained from the Japanese Collection of Research Bioresource (JCBR) Cell Bank, Osaka, Japan. These cell lines were cultured in F-12 Nutrient Mixture (Ham’s F-12) (Gibco/Invitrogen, Calsbald, CA) supplemented with 10% fetal bovine serum (Gibco/Invitrogen), 1% antibiotic-anti-mycotic (Gibco/Invitrogen) and 14 mM NaHCO_3_. Cells were incubated in a humidified incubator at 37 °C, 5% CO_2_.

CCA cells in 6 cm-dish were treated with Akt inhibitor, 1 µM of MK2206 (Sigma Aldrich, St. Louis, MO) or Erk inhibitor, 10 µM of PD98059 (Cell Signaling Technology, Danvers, MA) for 6 and 12 h. Cells had been washed before cell lysates were prepared for Western blotting analysis. For short-time stimulation of EGF, CCA cells (2 × 10^5^ cells/well) were seeded into a 6-well plate. After 24 h treatment of siFUT1 or UEA-I, CCA cells were cultured in a serum-free Ham’s F-12 media for 2 h then stimulated with 100 ng/ml of EGF (Peprotech, Rocky Hill, NJ) in serum free media for 10 min. Cells were washed immediately with cold PBS and harvested with lysis buffer for Western blotting analysis.

### UEA-I histochemistry

The lectin histochemistry was performed as previously described^11^. Briefly, non-specific background was blocked with 0.5% periodate-treated BSA (tBSA) for 30 min at room temperature. The tissue sections were then incubated with 1:100 biotinylated UEA-I (Vector laboratories, Burlingame, CA) at room temperature for 2 h and with 1:100 streptavidin-conjugated HRP (Invitrogen, Camarillo, CA) for 40 min. The peroxidase activity was developed by diaminobenzidine tetrahydroxychloride solution (DAB; Dako, Glostrup, Denmark) and counterstained with Mayer’s hematoxylin (Bio-optica; Milan, Italy). The TFG of each tissue section was scored under light microscopy according to the Allred scoring system^50^. In brief, the percentage of positive stained cells were graded as 0 = negative, 1–25% = 1, 26–50% = 2, 51–75% = 3 and >76% = 4. The intensities of the stained cells were scored as 1 = weak, 2 = moderate, 3 = strong. The TFG score was calculated as frequency x intensity and classified into low and high according to the medians of the TFG scores.

### UEA-I cytofluorescent staining

CCA cells (5 × 10^4^ cell/well) were seeded into a Matrigel-coated slide chamber and incubated at 37 °C, 5% CO_2_, overnight. After transfection with siFUT1, CCA cells were fixed with 4% paraformaldehyde at room temperature for 30 min and the non-specific binding was blocked with 0.5% tBSA. Cells were then incubated with 1:500 biotinylated UEA-I at 4 °C, overnight, and with 1:500 streptavidin-conjugated Alexa Fluor 488 (Invitrogen, Carlsbad, CA) at room temperature in the dark for 1 h. Nuclei were stained with 1:2,000 Hoechst 33342 (Invitrogen, Eugene, OR). The fluorescent images were graded with a fluorescence microscope (ECLIPSE Ni-U; Nikon, Tokyo, Japan).

### Cell aggregation assay and UEA-I treatment

As lectin can cause cell aggregation, the appropriate UEA-I concentration for UEA-I treatment was first optimized. KKU-213 cells (5 × 10^4^ cells/well in a 24-well plate) were incubated with a two-fold dilution of 100 µg/ml unconjugated UEA-I (Vector laboratories, Burlingame, CA) in serum-free Ham’s F-12, 1 h. The aggregated cells were observed under light microscopy (Fig. S1). The highest UEA-I concentration that did not generate cell aggregation was used for UEA-I treatment. For migration and invasion assays, CCA cells (3 × 10^5^ cells/well) were plated into a 6-well plate for 24 h and then treated with 5 µg/ml UEA-I for 24 h before the migration and invasion assays were performed in the presence of UEA-I. For the wound scratching assay, wound closures were determined in the presence of 5 µg/ml UEA-I. Experiments performed in the absence of lectin were used as controls.

### Proliferation assay

CCA cell lines; KKU-213 (6 × 10^3^ cells/well) and KKU-214 (4 × 10^3^ cells/well) were seeded in a 96-well plate for 16 h. Cells were transfected with siFUT1 or siControl and cultured for 24, 48 and 96 h. Cell proliferation was measured using the sulforhodamine B (SRB) colorimetric assay^51^. At the end of incubation time, cells were fixed with cold 10% TCA in 1% acetic acid at 4 °C for 30 min. Fixed cells were stained with 0.4% SRB in 1% acetic acid at room temperature for 30 min and destained with 1% acetic acid. The stained cells were dissolved with 10 mM Tris-base solution (pH 10.5) at room temperature for 30 min on a shaker. The optical density was measured at 540 nm using a microplate reader (SUNRISE, Groedig, Austria).

### Wound scratching assay

CCA cells (2–2.5 × 10^4^ cells/well) were seeded into a 24-well plate and cultured overnight to reach 80–90% confluent cells. The cell monolayer was scratched and washed with PBS twice. The wound closure was detected at 0, 6, 12 and 24 h under a light microscope. The migrated distance was calculated by subtracting the distance between the width of wound at the indicated time from that at time 0 h then divided by the width of wound at time 0 h.

### Migration and invasion assay

Migrated and invaded cells were determined using Transwell^®^ Chambers (8.0 µm-pore size; Corning, Lowell, MA) as described previously^52^. The insert was pre-coated with 100 µl of 0.4 mg/ml of Matrigel overnight before seeding cells for the invasion assay. After 24 h of siRNA transfection, cells were resuspended in serum free media and seeded into the upper chamber. Cells (4 × 10^4^ cells) were allowed to migrate or invade to the lower chamber containing completed media; 9 h for KKU-213 and 24 h for KKU-214. The migrated and invaded cells were fixed and stained with 0.5% crystal violet as described previously. The crystal violet-stained cells were counted under the light microscope using a 10X objective lens, 9 microscopic fields/insert.

### Adhesion assay

A 48-well plate was pre-coated with 50 µg/ml of Matrigel, overnight. After blocking with 2% BSA in PBS for 1 h, siRNA-transfected cells (3 × 10^4^ cells/well for KKU-213 and 2 × 10^4^ cells/well for KKU-214) were seeded and allowed to adhere for 2 h at 37 °C, 5% CO_2_. Non-adhered cells were carefully removed. The adhered cells were fixed with 4% paraformaldehyde for 30 min and stained with 0.5% crystal violet. The stained cells were dissolved with 2% SDS and the absorbance was measured at 540 nm.

### Determination of FUT1 expression by quantitative real-time PCR

Total RNA was extracted from CCA cell lines using Trizol reagent (Ambion, Carlsbad, CA) and converted to cDNA by a high capacity cDNA reverse transcription kit (Applied Biosystems, Foster, CA). All steps were done according to the instructions of the manufacturer. The PCR reactions were performed using 40 ng of cDNA, 2.5 µM of primer mix (F + R) and 2X LightCycle 480^®^ SYBR green I master (Roche Diagnostics, Mannheim, Germany). The conditions of amplifications were setup according to the LightCycle480^®^ instrument protocol as 95 °C for 5 min, followed by 35 cycles of 95 °C for 10 sec, 60 °C for 10 sec and 72 °C for 10 sec and finally 40 °C for 10 sec. The expression of FUT1 mRNA was quantitated from the triplicated samples using LightCycle480^®^ Relative Quantification Software (Roche Diagnostics). β-actin was used as an internal control. The primer sequences of FUT1 and β-actin were: FUT1 as F: 5′-TGAGGGATCACTGCCAAAATG-3′, R: 5′TCTTGGCAGTTTATGAGCTTTAAAAA-3′ and β-actin as F: 5′TCGTGCGTGACATTAAGGAG-3′, R: 5′GAAGGAAGGCTGGAAGAGTG-3′.

### Transient knockdown of FUT1 by siRNA

Specific siRNA to FUT1 mRNA was used to suppress FUT1 expression in CCA cells. The sequences of siFUT1 were: sense strand; 5′-GUAAUCUUCUUCCUCCAUA-3′ and anti-sense strand: 5′-UAUGGAGGAAGAAGAUUAC-3′. CCA cells (2 × 10^5^ cells/well) cultured in a 6-well plate overnight were transfected with 100 pmole of siFUT1 using 2 µg/µl of Lipofectamine 2000 according to the recommendation of manufacturer (Invitrogen, Carlsbad, CA). Cells were cultured in HAM’s F-12 media with 10% FBS and harvested after 24 h. Control experiments were performed using siControl (Negative control siRNA#1027310, Qiagen, Chatsworth, CA).

### SDS-PAGE and western blotting analysis

Cells were lysed with lysis buffer (1% NP-40, 0.5% Na-deoxycholate, 0.1% SDS, 2 mM EDTA, 150 mM NaCl and 50 mM Tris-HCl pH 7.4) containing phosphatase- and protease-cocktail inhibitors (Roche, Mannheim, Germany) and incubated at 4 °C for 15 min. Cell lysates were obtained after centrifugation at 12,000 xg, 4 °C for 15 min and total protein was quantified by the Quick Start^TM^ Bradford protein Assay (Bio-Rad, Hercules, CA). Protein lysates (20 µg/well) were separated in a 10% SDS-PAGE^53^ and transferred on to a PVDF membrane (Amersham^TM^, Buckinghamshire, UK) using Bolt and Mahoney transferring buffer^54^. For S100A4, the nuclear fraction was extracted according to Andrews *et al*.^55^. Nuclear lysate of 20 µg/well was loaded onto a 15% SDS-PAGE, and histone H1 was used as the internal control.

The antibodies used for Western blot were anti-β-actin (A5441) (Sigma Aldrich, St. Louis, MO), anti-Erk1/2, anti-p-Erk1/2, anti-STAT3 (C20), anti-p-STAT3 (Y705), anti-p-STAT3 (S727) (S727-R) (Santa Cruz Biotechnology, Santa Cruz, CA), anti-E-cadherin, anti-Cludin-1, anti-Slug, anti-N-cadherin, anti-Vimentin and anti-EGFR (Y1068; D7A5) (Cell Signaling Technology, Danvers, MA), S100A4 (Abnova, Taipei, Taiwan). The signal was developed by the ECL^TM^ Prime Western Blotting Detection System (Amersham^TM^, Buckinghamshire, UK). The imaging was obtained using an ImageQuant LAS 4000 mini image analyzer and analyzed by ImageQuant^TM^ TL analysis software (GE Healthcare, Buckinghamshire, UK).

### Statistical analysis

Statistical analysis was performed using the SPSS version 17.0 (SPSS Inc., Chicago, IL) and GraphPad Prism^®^ 5.0 software (GraphPad Software, Inc., La Jolla, CA). Survival analysis was performed using Kaplan-Meier statistics with *P*-values computed by log-rank tests. A subsequent backward stepwise multivariate analysis using a Cox regression model was used to signify the variables that were associated with survivals of patients with CCA. The chi-square test was used to compare the differences in the clinical features of patients. The two-tail Student’s t-test was used to compare the differences between treatments and controls. At least two independent experiments were performed. All quantitative data was mean ± SD of the triplicate samples from one of two independent experiments. *P* < 0.05 was considered as the statistical significance.

## Supplementary information

Supplementary Table 1 and Figures
