# Supplementary material for: Terminal fucose mediates progression of human cholangiocarcinoma through EGF/EGFR activation and the Akt/Erk signaling pathway
Source: Sci Rep. 2019 Nov 21;9:17266. doi: 10.1038/s41598-019-53601-8 (PMC6872661; doi:10.1038/s41598-019-53601-8)

Terminal fucose mediates progression of human cholangiocarcinoma through EGF/EGFR activation and the Akt/Erk signaling pathway

Somsiri Indramanee[1, 2], Kanlayanee Sawanyawisuth[1, 2], Atit Silsirivanit[1, 2], Paweena Dana[1, 2], Chatchai Phoomak[1, 2], Ryusho Kariya[4], Nathakan Klinhom-on[1, 2], Supannika Sorin[1, 2], Chaisiri Wongkham[1, 2], Seiji Okada[4*], Sopit Wongkham[1, 2, 3*]

[1]Department of Biochemistry, Faculty of Medicine, Khon Kaen University, Khon Kaen, Thailand, 40002

[2]Cholangiocarcinoma Research Institute, Khon Kaen University, Khon Kaen, Thailand, 40002

[3]Center for Translational Medicine, Khon Kaen University, Khon Kaen, Thailand, 40002

[4]Division of Hematopoiesis, Center for AIDS Research, Kumamoto University, Kumamoto, Japan, 860-0811

Supplementary Table 1. Univariate analysis of TFG expression and clinical features of patients with cholangiocarcinoma

**Clinical feathers**	**Expression of TFG**			
	**Total (n)**	**Low**	**High**	***P*-value**
Age (years)				
≤ 56	41	24	17	0.447
> 56	38	19	19	
Sex				
Male	54	31	23	0.435
Female	25	12	13	
Histological type				
Papillary type	27	17	10	0.273
Non-papillary type	52	26	26	
Tumor size				
≤ 7 cm	50	24	26	0.132
> 7 cm	29	19	10	
Tumor stage				
I-III	30	18	12	0.437
IVA and IVB	49	25	24	
Regional lymph node metastasis				
No	38	23	15	0.634
Yes	31	17	14	

Supplementary Figure 1. Optimization of UEA-I concentration on cell aggregation.

The cell aggregation test was performed as described in materials and methods. (A) KKU-213 cells were treated with various concentrations (0-100 µg/ml) of UEA-I for 1 h. Cell aggregation was observed under light microscopy. Aggregated cells were first observed at 6.25 µg/ml of UEA-I. (B) Cells were incubated with 5 µg/ml UEA-I for 9-24 h. No aggregated cells were detected during the observed time. Scale bar = 100 µm.

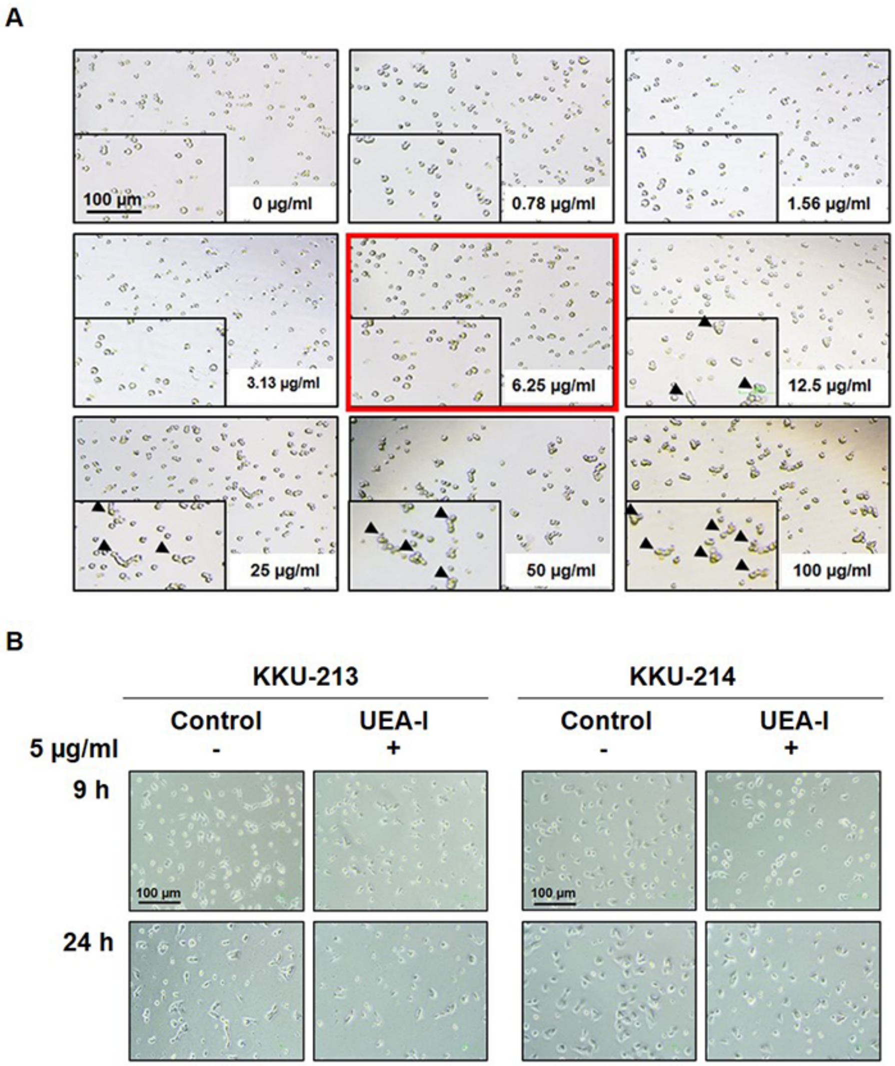

Supplementary Figure 2A. The full-length blots of Figures 5A

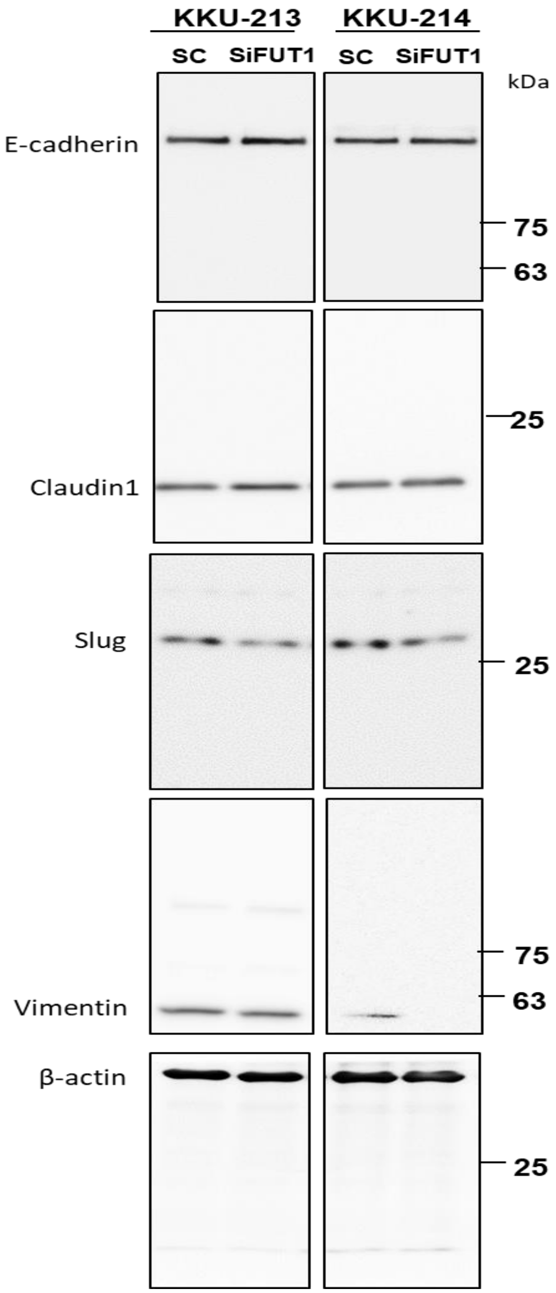

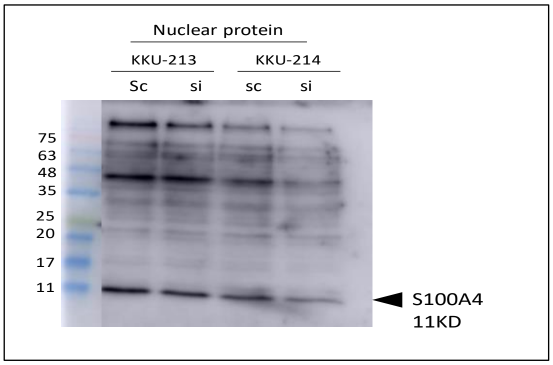

Supplementary Figure 2B. The full-length blots of Figure 5B

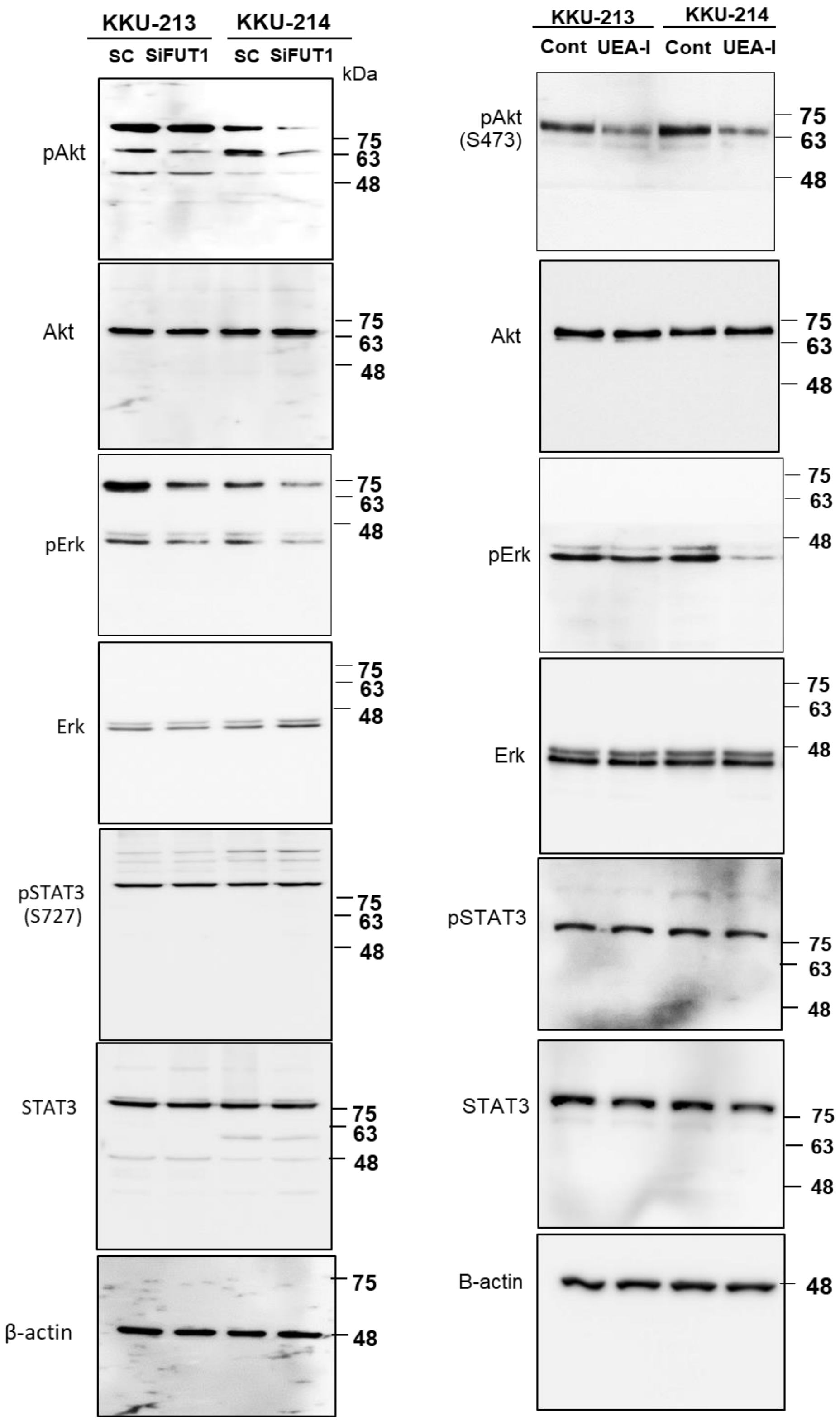

Supplementary Figure 2C. The full-length blots of Figure 5C

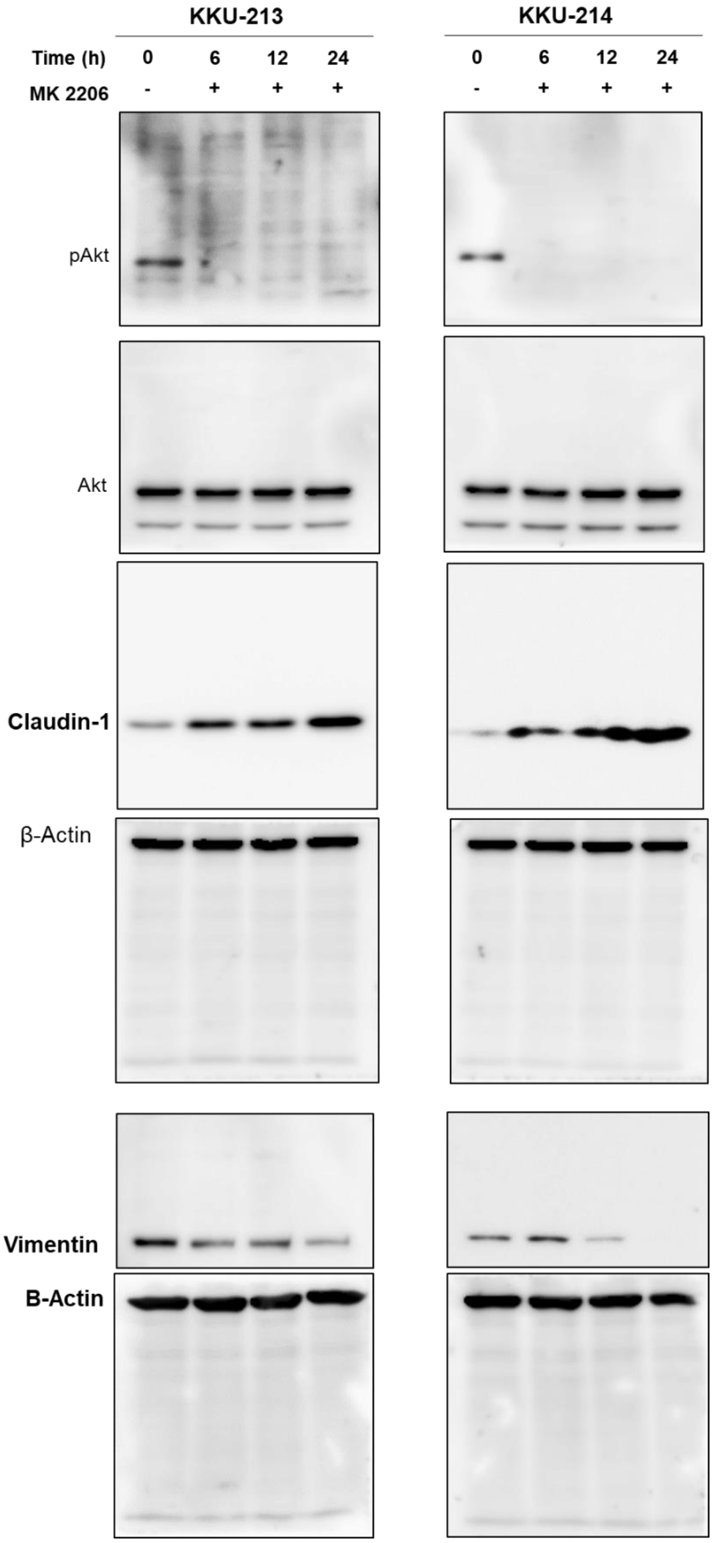

Supplementary Figure 2D. The full-length blots of Figure 5D

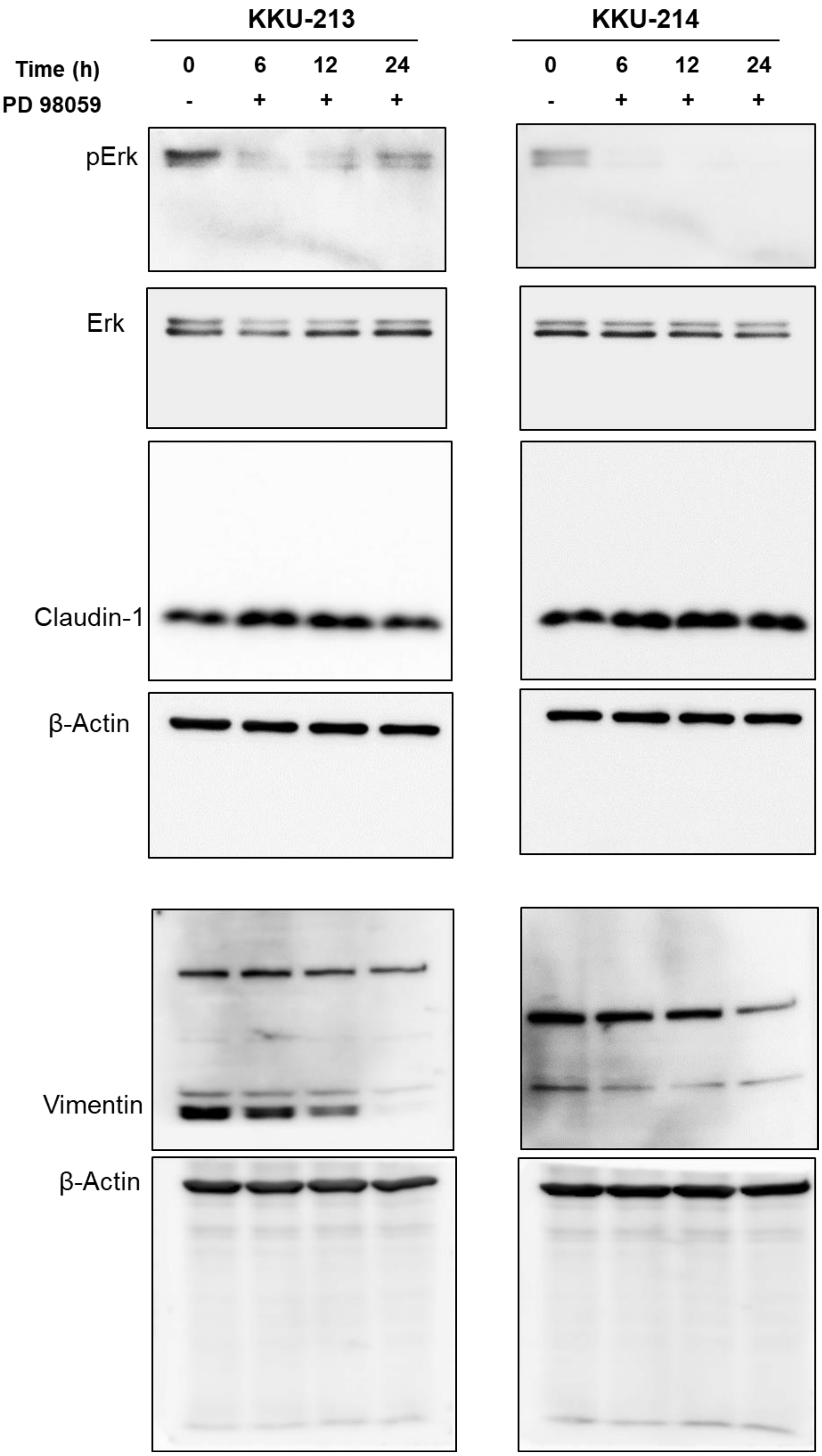

Supplementary Figure 3A. The full-length blots of Figure 6A

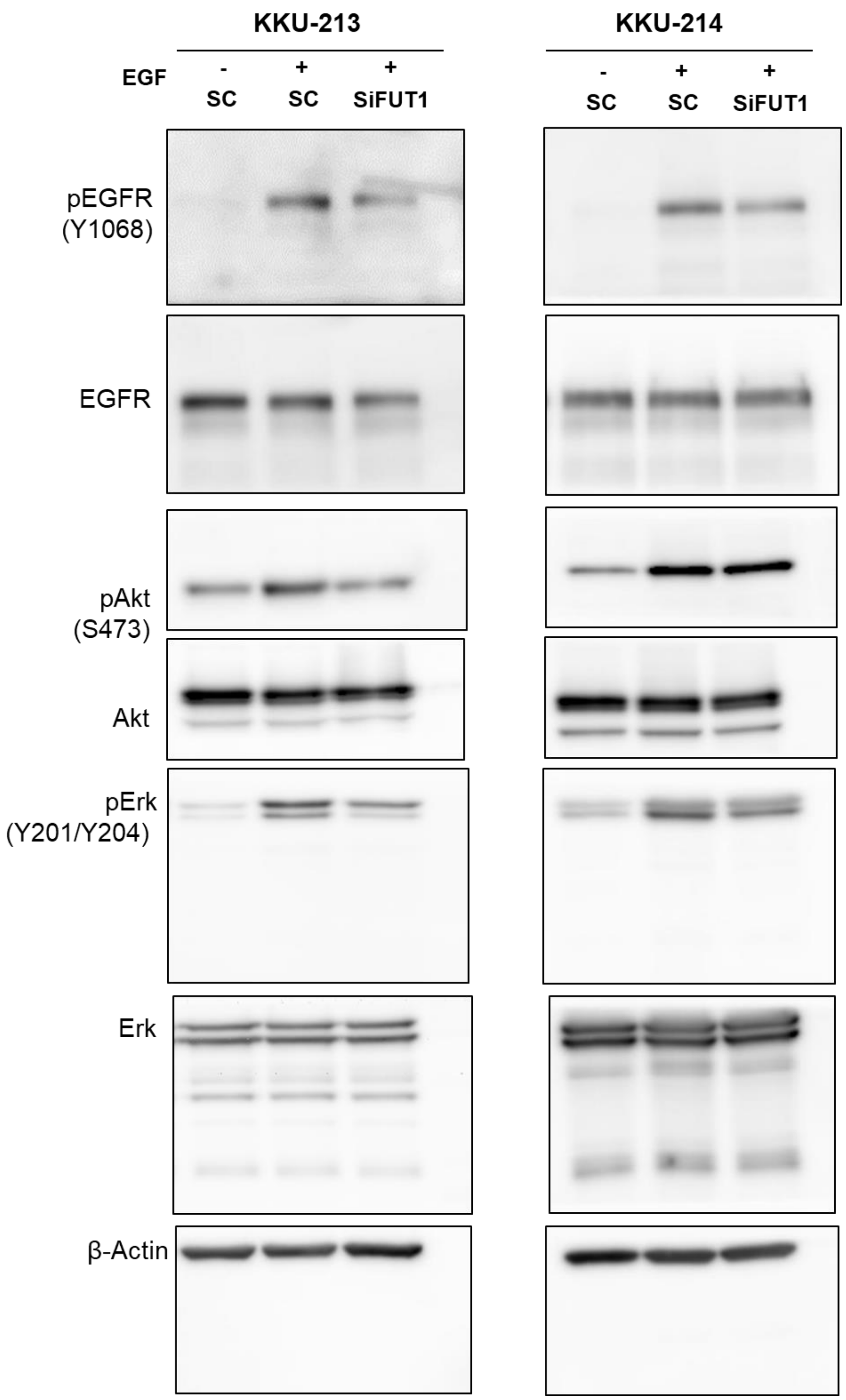

Supplementary Figure 3B. The full-length blots of Figure 6B

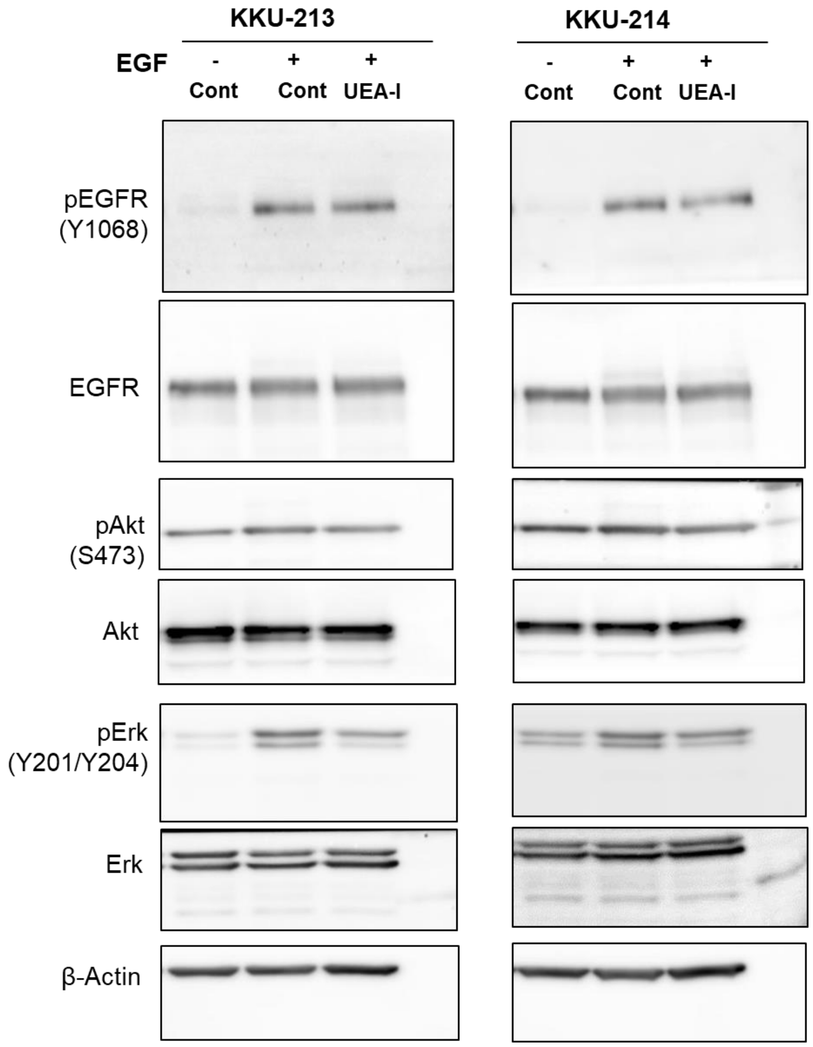

Supplement: Supplementary file 1 — Supplementary Table 1 and Figures [file 41598_2019_53601_MOESM1_ESM.pdf]
